# Creation of stable *Pseudomonas aeruginosa* promoter–reporter fusion mutants using linear plasmid DNA transformation

**DOI:** 10.1186/s13104-016-2130-3

**Published:** 2016-06-24

**Authors:** Ping Chen, Kai P. Leung

**Affiliations:** Dental and Craniofacial Trauma Research and Tissue Regeneration, US Army Institute of Surgical Research, JB Fort Sam Houston, 3650 Chambers Pass, Bldg 3610, San Antonio, TX 78234-6315 USA

**Keywords:** *Pseudomonas aeruginosa*, Promoter activity, Green fluorescent protein, Linear DNA transformation

## Abstract

**Background:**

*Pseudomonas aeruginosa* is an important opportunistic human pathogen that is commonly encountered clinically in different types of infections. Reporter-gene systems and construction of mutants defective in specific functions are useful tools for studying the cellular physiology and virulence of this organism. The common mutant construction process requires constructing target alleles into large size suicide vector(s) for transformations, and extra steps involved in resolving merodiploids. Here we describe a new approach using linearized plasmid transformation for creating a green fluorescent protein (GFP) reporter gene system to study promoter activities in *P. aeruginosa*.

**Findings:**

We successfully created promoter–reporter fusion plasmids for studying the promoter activity of virulence genes in *P. aeruginosa*. The promoter of exoenzyme S (a virulence factor) was used in preparation of these fusion plasmids. These fusion plasmids were linearized and used directly to transform *P. aeruginosa*. Stable *P. aeruginosa* chromosomally integrated promoter–reporter fusion mutants were obtained. We demonstrated that the promoter of Exoenzyme S gene was activated when *P. aeruginosa* was grown in a biofilm state, as evidenced by the expression of GFP in these biofilm cells.

**Conclusion:**

Direct transformation with linearized plasmid DNA provides another avenue to create *P. aeruginosa* mutants. This new approach eliminates the use of suicide vector(s) for creating *P. aeruginosa* mutants, and thus speeds up the process mutant construction.

**Electronic supplementary material:**

The online version of this article (doi:10.1186/s13104-016-2130-3) contains supplementary material, which is available to authorized users.

## Background

The ubiquitous Gram-negative *Pseudomonas aeruginosa* is an important opportunistic human pathogen. This organism has a large genome of 6.3 million nucleotides [1], and causes both life-threatening acute infections as shown in burn patients, and chronic lung infections as in cystic fibrosis patients [2]. *P. aeruginosa* lives in two different life styles, planktonic and biofilm, which are thought to be associated with acute infections and chronic infections, respectively. The expression of virulence in *P. aeruginosa*, which can be influenced by environmental signals, is tightly controlled by complex regulatory networks. About 8.4 % of predicted *P. aeruginosa* genes are involved in regulation, which is among the highest proportion of predicted regulatory genes observed in sequenced bacterial genomes [1]. Therefore, it is important to understand the regulatory pathways controlling the expression of virulence in *P. aeruginosa* under different conditions and time. This can be accomplished by studying the promoter activities of virulence genes at both individual and global gene expression levels (such as RNA sequencing).

To study the promoter activity of a particular gene, the promoter is commonly fused to a reporter gene [such as green fluorescent protein (GFP) gene] in a replicative plasmid in order to obtain the highest levels of fluorescence. The resultant plasmid is introduced into the host bacterium, and the activities of the reporter gene are measured under the desired test conditions. To prevent loss of the plasmid, antibiotic is added into the growth medium to maintain the selection pressure. However, in some circumstances (such as polymicrobial interaction studies), the continuous presence of antibiotics to maintain the selection pressure is not desirable because some interacting strains could be susceptible to the antibiotic. In addition, antibiotics themselves can have untoward effect on gene expression circuits. Therefore, it is more preferable to have the fused promoter–reporter element stably integrated into the bacterial chromosome.

Creating stable chromosomal insertion/deletion mutants involves several steps [3, 4]. Firstly, target alleles that are often tagged with an antibiotic resistance gene are inserted into a suicide plasmid. Secondly, the resultant suicide plasmid is delivered into the host by electroporation or conjugation, followed by recombination of the plasmid-borne target into the chromosome. Finally, correct mutants are obtained by resolving the merodiploids that result from chromosomal integration of the suicide plasmid by a single crossover. This latter procedure uses a counter-selectable marker such as *Bacillus subtilis sacB* (sucrose counter selection). Some drawbacks of this conventional approach include: (1) difficulties encountered in *sacB* counter-selection due to issues of stringency; and (2) cloning difficulties due to the relatively large sizes of suicide plasmids themselves and few available cloning sites. In this report, we showed that *P. aeruginosa* mutants can be directly created by electroporation of high concentrations of linearized cloning plasmid DNA, eliminating the use of a suicide plasmid.

## Methods

### Growth conditions for bacterial strains and plasmids manipulations

*Escherichia coli* 5α strains used for subcloning and plasmid isolation were grown in Luria–Bertani (LB, 10 g/l of tryptone, 10 g/l of NaCl and 5 g/l of yeast extract) medium at 37 °C in the presence of the appropriate selective substance (10 μg/ml of Gentamicin), or in low salt LB medium (10 g/l of tryptone, 5 g/l of NaCl and 5 g/l of yeast extract) containing antibiotic zeocin (50 μg/ml) (Invitrogen, Carlsbad, CA). *E. coli* plasmid DNA isolations were carried out by the QIAprep Spin Miniprep Kit (QIAGEN, Valencia, CA). Routine procedures were employed for manipulation of DNA. Unless specified, *P. aeruginosa* PAO1 was grown in LB and clinical strains were grown in brain heart infusion (BHI).

### Promoter–reporter fusion plasmid constructions

**Fig. 1:**
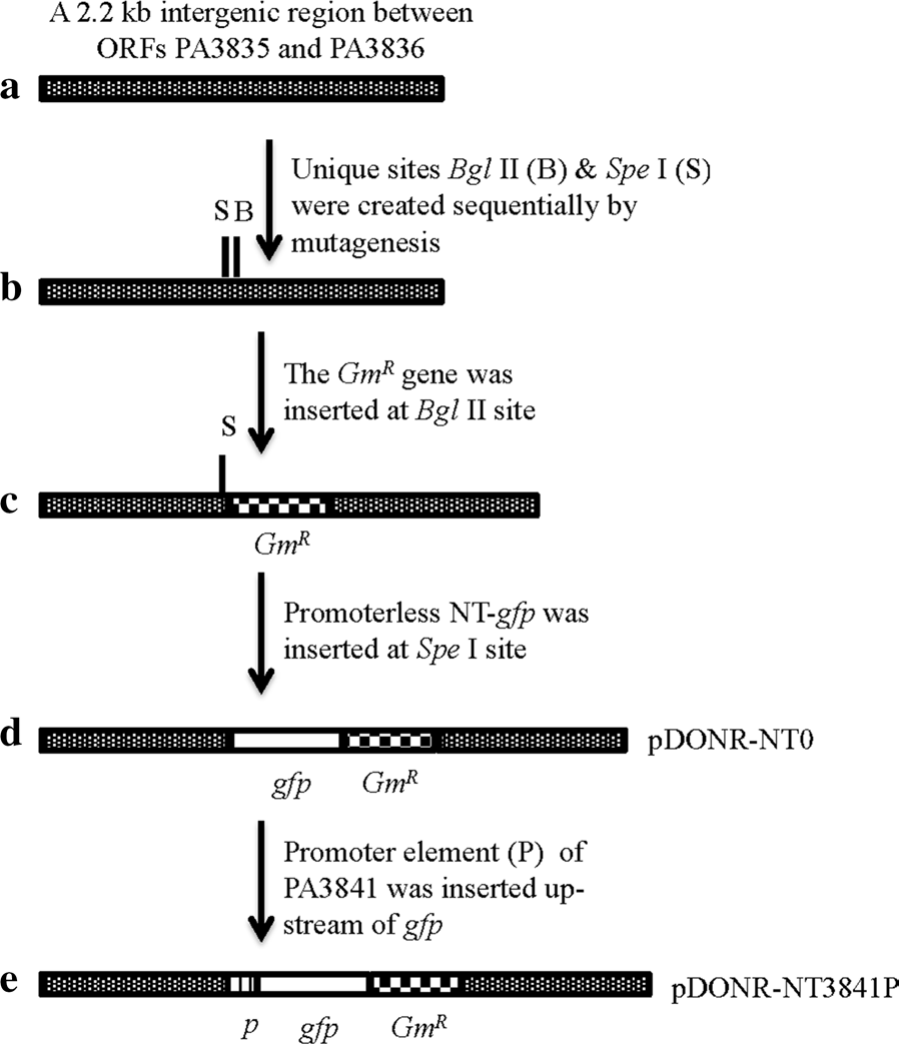
Schematic of inserting the fused promoter–reporter element into *P. aeruginosa*. **a** A roughly 2.2 kb intergenic region between ORFs of PA3835 and PA3836 was PCR amplified and cloned into vector pDONR/Zeo obtained from Invitrogen-Thermo Fisher Scientific (Grand Island, NY). **b** Two unique sites, *Bgl*II (B) and *Spe* I (S) were sequentially created by site-directed mutagenesis with the *Bgl*II site located in the* middle* of the intergenic region, and the *Spe*I site located at 10 bp upstream of the *Bgl*II site. **c** The gentamicin resistance gene (*Gm* ^*R*^) was amplified by PCR and inserted at the *Bgl*II site. **d** The long half-life version of *gfp* was inserted at the *Spe*I site, generating plasmid pDONR-NT0. **e** Promoter of PA3841was inserted upstream of *gfp* in pDONR-NT0, creating construct pDONR-NT3841P. The final plasmid was digested with *Xho*I, dephosphorylated, column purified and used to electroporate wild-type *P. aeruginosa* PAO1 according to a published method [6]. The promoter–reporter element was inserted into the chromosome by a double cross-over recombination event. Primers used were listed in Additional file 1: Table S1

Steps for inserting the fused promoter–reporter element into the *P. aeruginosa* chromosome are illustrated in Fig. 1. Briefly, an approximate 2.2 kb intergenic region between open reading frames (ORFs) of PA3835 and PA3836—a region without any transcription based on our unpublished RNA Sequencing (RNA-Seq) data—was PCR-amplified using primers (listed in Additional file 1: Table S1) containing *Xho*I sites, and cloned into cloning vector pDONR/Zeo from Invitrogen. Using the site-directed mutagenesis method, two unique sites, *Bgl*II and *Spe*I, were created sequentially with *Bgl*II site located in the middle of the intergenic region, and *Spe*I site located at 10 bp up-stream of the *Bgl*II site. Using plasmid pJQ200 (ATCC^®^77482) as a template, the gentamicin-resistance gene with its own promoter, was PCR amplified with *BamH*I site-containing primers, digested with *BamH*I, and inserted at the *Bgl*II site in the intergenic region. A long half-life version of *gfp* containing its own ribosome binding site with surrounding *rrnB* T1 terminator sequences was amplified by PCR from vector pPROBE-NT [5], and inserted at the *Spe*I site upstream of the gentamicin-resistance marker. This construct was designated as pDONR-NT0. It has three unique restriction enzyme cutting sites (*Xba*I, *Kpn*I & *EcoR*I) for cloning of any promoter fragment to be studied into the upstream of the reporter *gfp*.

The sequence of the test promoter was PCR amplified and inserted into upstream of the reporter *gfp* at the unique site(s) (*Xba*I, *Kpn*I & *EcoR*I). The resultant plasmid was digested with *Xho*I and dephosphorylated using shrimp alkaline phosphatase (rSAP) to prevent self-religation. The treated and linearized DNA was column purified using Wizard SV PCR clean-up mini-column from Promega (Madison, WI), and used directly for electroporation.

### *P. aeruginosa* transformation

*P. aeruginosa* electro-competent cells were prepared according to a previously published method [6]. Cells from 6 ml of an overnight culture grown in LB or BHI broth with an optical density of approximately 1.3 (λ = 600 nm) were harvested, washed, and re-suspended in 100 µl room temperature 300 mM sucrose. The cell suspension was mixed with approximately 5 µg Wizard SV PCR clean-up mini-column-purified linearized DNA. Transformation was carried out using an ECM630 Electro Cell Manipulator from BTX (Holliston, MA) in a 2 mm gap width electroporation cuvette with the following settings: 25 µF, 189 Ω, and 2.5 kV. Following the immediate addition of Super Optimal Broth (SOC) medium (0.6 ml) after electroporation, cells were transferred to a 17 × 100 mm style 14 ml polypropylene round-bottom tube, and incubated for 2 h at 37 °C with shaking at 150 rpm. The entire mixture was plated on four PIA (*Pseudomonas* isolation agar; Becton, Dickinson and Co.) plates, each containing 40 µg/ml gentamicin. After overnight incubation, colonies were analyzed for proper double crossover recombination by colony PCR based on the sizes of the PCR products.

### Growing *P. aeruginosa* biofilms in the bioflux system

The mid-log phase *P. aeruginosa* culture was passed through a 5-µm syringe filter (Pall Corporation, Ann Arbor, MI) to reduce the number of aggregates. For inoculation, the culture was adjusted to an optical density of 0.1 at the wavelength of 600 nm (OD_600_). The channels of a 48-well microplate from the Bioflux system (Fluxion Biosciences, South San Francisco, CA) were primed with media, followed by inoculation of each channel with 50 µl *P. aeruginosa* suspension (OD600_nm_ = 0.1). After 2 h of attachment, the channels were perfused with 50 % BHI medium at a shear flow of 0.55 dyn/cm^2^ to initiate the growth of biofilms. Biofilm formation and green fluorescence were monitored in real time using the LSM710 confocal microscope (Carl Zeiss MicroImaging, Thornwood, NY).

## Results and discussion

To study the promoter activities, we constructed a promoter–reporter fusion plasmid pDONR-NT0 (see Fig. 1). This plasmid contains three unique restriction enzyme cutting sites (*Xba*I, *Kpn*I & *EcoR*I) for cloning of any promoter fragment to be studied into the upstream of the long half-life version *gfp* reporter. Gentamicin-resistance marker was used for selection. The insertion site was chosen to be located at the intergenic region between open reading frames of PA3835 and PA3836—a region without any transcription based on our unpublished RNA Sequencing (RNA-Seq) data. Basic local alignment search tool (BLAST) results showed that although the sequence could vary, most, if not all, *P. aeruginosa* strains have this intergenic region. Because this region is non-coding and lack of transcription activity; therefore, this insertion site is not likely to interfere with other gene functions.

To prove the concept of transformation using linearized plasmid DNA, we used the promoter element of Exoenzyme S structural gene, a type III secretion effector and an important virulence factor for *P. aeruginosa* [7]. We amplified this promoter by PCR and cloned it into pDONR-NT0 (see Fig. 1) to create pDONR-NT3841P. We transformed 5 µg of each of these linearized plasmids into *P. aeruginosa* PAO1 competent cells. The rational for using 5 µg total DNA is that this amount has consistently yielded enough transformants for downstream characterization in our preliminary determinations. In addition, it has been shown that the number of recombinants generated from 5 µg total DNA is close to the maximum number of transformants that can be obtained in other Gram-negative bacteria [8]. Eight and twelve transformants were obtained from the pDONR-NT0 and pDONR-NT3841P transformation respectively. PCR analyses confirmed that about 50 % of the transformants were correct double crossover recombinants (Table 1). The recombinants were designated as *P. aeruginosa* NT0 and NT3841P respectively. There was no difference in growth patterns between the double crossover recombinants obtained *vs.* wild-type *P. aeruginosa* PAO1 (data not shown). The incorrect double crossover recombinants were spontaneous gentamicin-resistant mutants and single crossover recombinants that were likely resulted from the incomplete enzyme digestions.

**Table 1:** Recombination frequencies of *P. aeruginosa* PAO1 transformation using linearized plasmid DNA

Constructs^a^	# Of transformants	# Of correct recombinants	Correct ratio (%)^b^
pDONR-NT0	8	4	50
pDONR-NT3841P	12	6	50
pDONR-NT45P	7	3	42.8
pDONR-NT3194P	10	5	50
pDONR-ASV0	7	3	42.8
pDONR-ASV3841P	10	4	40
pDONR-ASV45P	11	4	36.4
pDONR-ASV3194P	12	6	50
pDONR-ASV4228P	7	3	42.8

^a^ *NT* long half-life version of GFP. *ASV* short half-life (110 min) version of GFP. *0* no promoter element. *P* promoter element from that particular ORF (e.g. 3841P—promoter from ORF PA3841)

^b^ *Ratio* # of correct recombinants per # of transformants

**Fig. 2:**
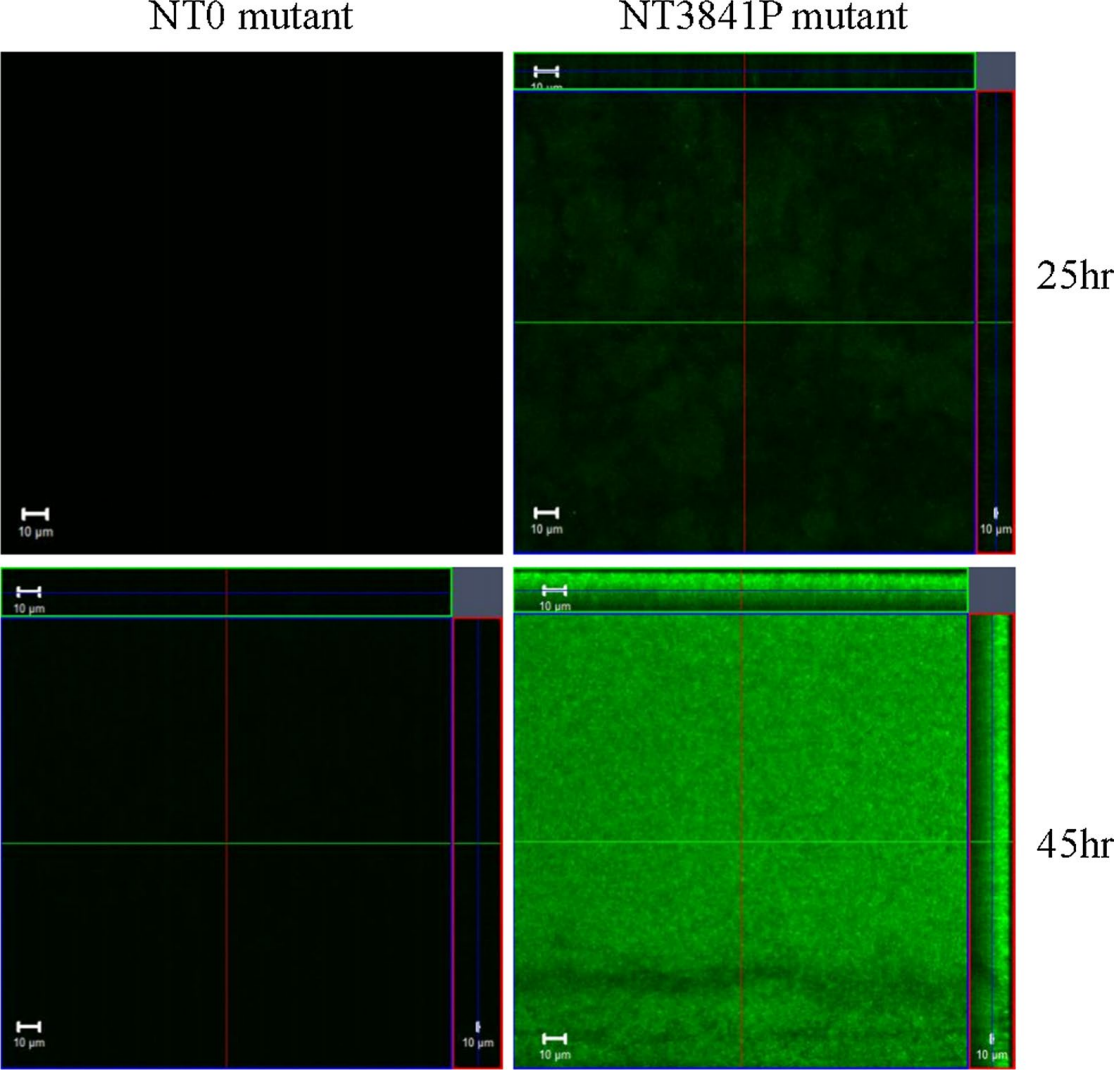
The promoter of Exoenzyme S gene (PA3841) was activated when *P. aeruginosa* cells were in the biofilm state. Recombinants *P. aeruginosa* NT0 & NT3841P were grown at 37 °C in a 48-well microplate Bioflux system (Fluxion Biosciences, South San Francisco, CA) with a shear flow of 0.55 dyn/cm^2^ using 50 % BHI (Brain Heart Infusion) medium. Images were captured after 25- and 45-h growth using a confocal microscope (LSM710; Carl Zeiss MicroImaging, Thornwood, NY) with the excitation wavelength set at 488-nm.* Left panels* and* right panels* are representative fluorescence images of NT0 and NT3841P mutants respectively. The expression of GFP was increased in the biofilms formed by the NT3841P recombinant, but not in the biofilms formed by the control NT0 recombinant. *Bars* represent 10 µm

To demonstrate the effectiveness of the promoter–reporter fusion system created, we compared the expression of green fluorescent protein between recombinant strains NT0 (promoterless control) and NT3841P which contained the promoter of exoenzyme S gene. Our transcriptome profiling (RNAseq) showed that the promoter of exoenzyme S gene was activated when *P. aeruginosa* grew in a biofilm. Indeed, as compared to the promoterless control, we observed increased fluorescence from the NT3841P recombinant grown as biofilms in the Bioflux System (Fig. 2). Although biofilm formation and type III secretion have been shown to be reciprocally regulated in some publications [9], biofilms and type III secretion are not mutually exclusive in *Pseudomonas aeruginosa.* Under some growth condition(s), the type III effector proteins (such as ExoS and ExoT) were expressed in biofilm cells, but not in the planktonic cells [10]. The discrepancy may be caused by different growth conditions used in the studies. The Bioflux system that we used to grow biofilm is a continuous-flow system which more closely mimics the in vivo conditions. Our results are in agreement with that of Mikkelsen et al., showing increased ExoS expression in biofilm cells grown in a continuous-flow system [10].

To further confirm the effectiveness of the linearized plasmid DNA transformation approach, we transformed *P. aeruginosa* PAO1 with other constructs containing the promoter of a hypothetical protein (PA45) gene, the promoter of a phosphogluconate dehydratase (PA3194) gene, and the promoter of pyochelin (PA4228) gene using different versions of *gfp* reporter genes. For each transformation, although there are variations in transformation efficiencies, we were able to obtain correct double crossover recombinants of these different constructs (Table 1). However, we were not able to monitor the activities of these promoters using our constructed system, possibly due to the weakness of the promoters and/or the test conditions are not optimal.

To determine if we can create deletion mutant using this linearized plasmid DNA transformation approach, a small none-coding regulatory RNA deletion plasmid was constructed (by Dr. Rajasekh Karna and Dr. Christine Miller in our laboratories) on the pCR2.1 vector backbone. The plasmid was linearized and transformed into *P. aeruginosa* PAO1 competent cells. Two hundred and eight gentamicin-resistant transformants were obtained. PCR analyses of randomly picked eight transformants showed that all were correct double crossover deletion mutants. We also used this linearized plasmid to transform a highly virulent clinical strain *P. aeruginosa* strain 12-4-4-59 [11]. We obtained eight correct double crossover deletion mutants. This result suggests that this linearized plasmid DNA transformation approach is not limited to PAO1 strain only.

Recombination efficiency could be influenced by the size of both the flanking region and the inserted non-homologous element, and by the surrounding sequence contexts. As the size of the inserted non-homologous DNA increases, the efficiency decreases exponentially; and once it reaches about 6 kb, the recombination efficiency is not detectable [12]. In contrast, as the flanking region size increases, the recombination efficiency increases. However, once the flanking region size reaches 1 kb, the recombination efficiency is not further improved [12]. In our case, the sizes of both flanking regions are ~1 kb, whereas the inserted non-homologous DNA is ~3 kb. Therefore, the low recombination efficiency observed is not likely due to the length of the flanking regions, but more likely due to the large size of the inserted element. The fact that we obtained more deletion mutants supports this. We were not able to improve the transformation efficiency by growing *P. aeruginosa* at 42 °C either for overnight or just for 2 h before performing the transformation. The presence of the vector in the total electroporated DNA preparation should not contribute to the observed low recombination efficiency either. In contrary, it may enhance recombination rates by saturating endogenous nucleases. It has been shown that the presence of carrier oligonucleotides increased the frequencies of recombination in both Gram-negative bacteria [8] and yeast [13].

Our results showed that *P. aeruginosa* mutants could be generated by electroporation of high concentrations of linearized cloning plasmid DNA. It has been reported that *P. aeruginosa* mutants could be created by electroporation of high concentrations of linear DNA, such as chromosomal DNA [6], PCR fragments [14], and single stranded synthetic oligonucleotides [15]. Compared to other forms of linear DNA, this linear plasmid DNA approach has two advantages: Firstly, this approach is not limited to any particular vector. Thus, it would be more convenient to manipulate a target region due to wider selections of vectors. Secondly, because cloning vectors are usually medium-to-high copy number plasmids, it is easier to obtain large quantities of plasmid DNA needed for the transformations. Compared to the conventional mutant generating approach that requires the use of suicide vector(s), this linear plasmid DNA approach eliminates the difficult subcloning steps into the suicide vector and the time consuming steps of resolving merodiploids resulted from single crossover events. One limitation of using linear plasmid DNA is the low transformation efficiencies. However, the number of correct recombinants obtained is still more than needed for downstream characterization.

## Conclusion

Direct transformation with linearized plasmid DNA provides another avenue to create *P. aeruginosa* mutants. This new approach eliminates the use of suicide vector(s) for creating *P. aeruginosa* mutants, and thus facilitates the study of this opportunistic pathogen.

## Additional file

10.1186/s13104-016-2130-3 Primers used in this study.
