# Supplementary material for: Creation of stable Pseudomonas aeruginosa promoter–reporter fusion mutants using linear plasmid DNA transformation
Source: BMC Res Notes. 2016 Jun 24;9:322. doi: 10.1186/s13104-016-2130-3 (PMC4921012; doi:10.1186/s13104-016-2130-3)
Supplement: Supplementary file 1 [file 13104_2016_2130_MOESM1_ESM.docx]

Primer sequence	Purpose	restriction site
5'-GGGGACAAGTTTGTACAAAAAAGCAGGCTCGCTCGAGCAACCAACTAAAGCAACTATGC-3′	clone PA3835 & PA3836 intergenic region into vector pDONR/zeocin	*Xho* I
5′-GGGGACCACTTTGTACAAGAAAGCTGGGTCCCTCGAGAAGGTCATCGGTTTCGAGCCCAAC-3	clone PA3835 & PA3836 intergenic region into vector pDONR/zeocin	*Xho* I
5′-CACACTCATGTGCGCTCATAGATCTATTAACGTGGAAATC-3′	Create Bgl II site in the PA3835 & PA3836 intergenic region	*Bgl* II
5′-GATTTCCACGTTAATAGATCTATGAGCGCACATGAGTGTG-3′	Create Bgl II site in the PA3835 & PA3836 intergenic region	*Bgl* II
5′-CATCCAGCGACCACACACTAGTGTGCGCTCATAGATCTATTAAC-3′	Create Spe I site in the PA3835 & PA3836 intergenic region before Bgl II site	*Spe* I
5′-GTTAATAGATCTATGAGCGCACACTAGTGTGTGGTCGCTGGATG-3′	Create Spe I site in the PA3835 & PA3836 intergenic region before Bgl II site	*Spe* I
5′-AATGGATCCACTTCGCTGCTGCCCAAGGTTG-3′	Amplify gentamycin marker from pJQ200	*BamH* I
5′-CGCGGATCCGTGACAATTTACCGAACAACTC-3′	Amplify gentamycin marker from pJQ200	*BamH* I
5′-CGTTCTAGACCTCGCCGGGAAAGTGTCCATG-3′	Clone PA3194 (phosphogluconate dehydratase) promoter	*Xba* I
5′-TATGAATTCTTGTTTTTACAACATAATCATCG-3′	Clone PA3194 (phosphogluconate dehydratase) promoter	*E. coR* I
5′-AGATCTAGAGCGAGAAAAAGCTGGTGGATG-3′	Clone PA3841 (exoenzyme S) promoter	*Xba* I
5′-TGAGAATTCTCCGCCAGTCTAGGAACG-3′	Clone PA3841 (exoenzyme S) promoter	*E. coR* I
5′-TGTTCTAGAGCAATGATGGTGATGGTCATCAG-3′	Clone PA4228 (pyochelin) promoter	*Xba* I
5′-CGTGAATTCGGTCGATTGCCATTGTTGGGAAATG-3′	Clone PA4228 (pyochelin) promoter	*E. coR* I
5'-GTACATAACCTTCGGGCATGGCAC-3′	To screen promoter incertion colonies	
5′-TAATACTAGTCACTATAGGGCGAATTGGAGCTG-3′	Clone GFP region from pPROBE-gfp plasmids	*Spe* I
5′-TTTCACTAGTGAAACAGCTATGACCATGATTACG-3′	Clone GFP region from pPROBE-gfp plasmids	*Spe* I
5′-ACGCATGATGGTGACGTTGTTGAC-3′	To confirme the double cross intergration in PAO1 chromosome	
5′-TCTGGTACTGCCACTTGAGGTTCTTG-3′	To confirme the double cross intergration in PAO1 chromosome	
5′-ACCTTCACGTCATACTTCCTGACTG-3′	To screen PA3835 & PA3836 intergenic region clones and the orientation of gentamycin cassette insertion and gfp insertion	
5′-CAGCTGCTAGAGGTCCAGCATTGC-3′		
5′- GTAATACTAGT**CACTATAGGGCGAATTGGAGCTG-3′**	Clone GFP region from pPROBE-gfp ASV plasmids	*Spe* I
5′-ACGGTACTAGTATAGGTACATTGAGCAACTGACTG-3′	Clone GFP region from pPROBE-gfp ASV plasmids	Spe I
5′-CAAGGGTACCTGCCTGGCTGCCACCGGTTG-3′	Clone 0045 (hypothetical protein) promoter into GFP-NT & pTurboR/GFP-PRL	*Kpn* I
5′-TCAGGGTACCTGGAAAAAAACGCTATCGACGCGGAG-3′	Clone 0045 (hypothetical protein) promoter into GFP-NT & pTurboR/GFP-PRL	*Kpn* I
